# Green Synthesis and Characterization of Nanographene–MnO Composite Nanoparticles for CO_2_ Capture: Adsorption Performance, Isotherm Analysis, and Reusability

**DOI:** 10.1021/acsomega.6c00581

**Published:** 2026-04-20

**Authors:** Sinan Kutluay, Nerihan Haskan

**Affiliations:** Chemical Engineering Department, 52971 Istanbul Technical University, Maslak, Istanbul 34469, Turkey

## Abstract

In this study, hybrid composite nanoparticles (MnO-NG/green) containing nanographene (NG) and manganese oxide (MnO) were produced by using an environmentally friendly synthesis approach, and their CO_2_ adsorption performance was investigated in detail. The conventional method was used to synthesize MnO-NG composites, which were then compared with MnO-NG/green composites prepared via green synthesis using bioextract from the hemp plant. The composite nanoparticles were structurally characterized using various analytical methods, including FTIR, XRD, SEM, TEM, EDX, and BET analyses. The surface morphology of the composites obtained through green synthesis demonstrated a more homogeneous and regular distribution of MnO nanoparticles on the NG surface. BET analysis revealed that the specific surface area of the MnO-NG/green composites was 629 m^2^/g, with a mean pore diameter of 4.65 nm. CO_2_ adsorption tests were conducted at 273 and 298 K under 1 bar, and it was determined that the MnO-NG/green composites achieved 5.81 and 4.94 mmol/g CO_2_ uptake capacities, respectively. These values were significantly higher than those of NG (2.59–2.07 mmol/g) and conventional MnO-NG (4.43–3.74 mmol/g) composites. Analysis of the adsorption isotherm models indicated that the experimental data were better fitted by the Langmuir model. The calculated isosteric heat of adsorption (*Q*_st_, 19.4–24.5 kJ/mol) suggests that the adsorption of CO_2_ by MnO-NG/green composites predominantly occurs via physisorption. The MnO-NG/green composites demonstrated high structural stability and thermal resistance in five-cycle reusability tests, showing a reuse efficiency of 97%. This study clearly demonstrates that the production of MnO-NG/green composite nanoparticles via a green synthesis offers a promising approach.

## Graphical abstract

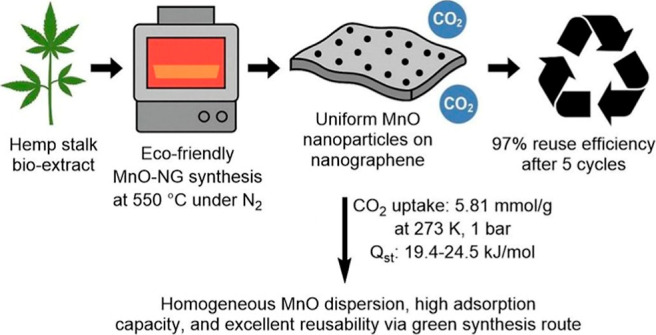

## 1 Introduction

Rapidly increasing greenhouse gas emissions in today’s world are cited as one of the primary causes of global climate change. The most widespread and impactful component of these emissions is carbon dioxide (CO_2_), which is released into the atmosphere in large quantities as a result of anthropogenic activities such as energy production, industrial activities, transportation, agriculture, and land use.^1–3^ CO_2_, in synergy with other greenhouse gases, causes heat retention in the atmosphere, leading to an increase in global temperature (global warming).^2^ This leads to a series of adverse environmental impacts, including melting polar ice caps, rising sea levels, and an increase in the frequency and severity of extreme weather events.^4^

The dramatic growth in human population and industrial activity over the past two centuries has led to a sharp increase in the level of CO_2_ emissions. Atmospheric CO_2_ concentration increased from 315 ppm in the 1960s to 419 ppm in 2023.^1^ A 2022 report by the Intergovernmental Panel on Climate Change (IPCC) stated that if the current upward trend in global temperatures and atmospheric CO_2_ levels continues, we can expect a 3.2 °C increase in global temperature and a 570 ppm increase in atmospheric CO_2_ by 2100 ^4^. Therefore, the development of technologies aimed at reducing and removing CO_2_ emissions from the atmosphere is of great importance.

In this context, carbon capture, utilization, and storage (CCUS) technologies are at the forefront.^5^ Among these technologies, adsorption-based approaches are particularly attractive due to their cost-effectiveness, ease of application, and regeneration potential.^6^ Solid surface adsorbent materials, in particular, have become a subject of widespread research because they enable selective and high-capacity CO_2_ capture. In the literature, materials such as organic frameworks (MOFs),^7^ covalent organic scaffolds (COFs),^8^ zeolites,^9^ activated carbons,^10^ polymers,^11^ metal oxides,^12^ silica, and biochar, as well as carbon-based nanostructures such as carbon nanotubes, graphene, and graphene oxide,^1,2,13^ have been investigated for this purpose.^4^

Graphene is a single-atom-thick, sp^2^-hybridized structure with a honeycomb arrangement of carbon atoms. It is one of the most remarkable nanomaterials due to its properties such as high specific surface area, excellent mechanical strength, and superior electrical and thermal conductivity.^2^ Thanks to these superior physicochemical properties, graphene and its derivatives are considered potential adsorbents in CO_2_ capture systems.^1,2^ However, the tendency for agglomeration due to π–π interactions between pure graphene layers may limit the use of this material alone. Therefore, surface modification of graphene with inorganic phases such as metal oxides allows this limitation to be overcome while improving functional properties.^14^

Manganese oxide (MnO) is a frequently preferred metal oxide in environmental applications due to its low toxicity, large surface area, high surface reactivity, and variable valence structure. Forming a nanocomposite by combining MnO with graphene oxide or reduced graphene oxide significantly increases the adsorption performance through a synergistic effect. Indeed, it has been reported in the literature that rGO-MnO_2_ nanocomposites exhibit significantly higher performance than conventional materials such as MOFs, zeolites, and commercial activated carbons, with a specific surface area of 710 m^2^/g and a CO_2_ adsorption capacity of 28.5 mmol/g.^15^ Furthermore, these composites exhibit stable reusability in the temperature range of 298–373 K for 20 cycles. Similarly, it has been shown that GO nanocomposites enriched with MgO nanoparticles exhibit strong chemisorption capacity toward CO_2_ at high temperatures (60–120 °C); this activity is due to the bidentate carbonate complexes formed between Mg^2+^–O^2–^ pairs and CO_2_.^16^ As demonstrated by these studies, metal oxide–graphene structures have the potential to be effective adsorbents. These adsorbents could be used in the CO_2_ capture technologies.

As environmental sustainability goals become more prevalent, interest in green synthesis methods has also increased. Green synthesis offers an alternative to traditional chemical reduction methods, which often require toxic reagents and high energy inputs. Green synthesis is a process that uses environmentally friendly resources such as plant extracts, microorganisms, and biopolymers to produce nanoparticles, reducing environmental impact.^17^ The production of various metal oxides, such as CuO, ZnO, Fe_3_O_4_, and MgO, through green synthesis and their use in environmental applications has been shown in the literature to be successful. However, research into MnO in graphene-based green-synthesized nanocomposites remains limited.

This study aims to synthesize nanographene (NG) and MnO composite nanoparticles using an environmentally friendly approach and to evaluate their CO_2_ adsorption performance. In addition, the sustainability of the hybrid structure was assessed in terms of cyclic use and application durability. Although various studies on CO_2_ adsorption of MnO-supported graphene nanocomposites exist, most are based on chemical synthesis methods or have been performed at a single temperature. Additionally, many lack an evaluation of cyclic usability, structural stability, and environmental impact. The production of MnO-NG composite nanoparticles via green synthesis therefore remains insufficiently explored. In this context, the homogeneous immobilization of MnO onto the NG surface through a green synthesis method and its influence on CO_2_ adsorption capacity represent an important research gap. This study addresses this gap by developing high-surface-area, thermally stable, and reusable MnO-NG composite nanoparticles using an environmentally friendly synthesis route. The reusability and temperature-dependent performance of the composites were evaluated through multicycle analyses, providing a scientific basis for potential laboratory- and industrial-scale applications. Despite growing interest in graphene–metal oxide hybrids for gas adsorption, no study has reported the synthesis or CO_2_ adsorption performance of MnO-nanographene (MnO-NG) composites. The only closely related work involves an rGO-MnO_2_ system,^15^ which differs fundamentally from the present study in both carbon structure and composite architecture. The high-surface-area nanographene skeleton used here differs structurally and chemically from conventional GO- or rGO-based systems. Furthermore, the use of a hemp-derived bioextract as a reducing and stabilizing agent enabled controlled and homogeneous MnO deposition, an approach rarely explored for CO_2_ adsorption. The combined evaluation of adsorption capacity, temperature-dependent behavior, isosteric heat, and multicycle stability reveals the decisive role of active site accessibility and interfacial chemistry. These aspects demonstrate the scientific originality of the MnO-NG/green composite developed in this study.

## 2 Experimental Section

### 2.1 Materials

All chemicals used in the experimental studies were obtained in analytical grade and were used directly without the need for further purification. Manganese oxide (MnO) used in this study was obtained from a manganese­(II) nitrate tetrahydrate (Mn­(NO_3_)_2_·4H_2_O) precursor compound with 99% purity in a laboratory environment to be used in the synthesis of MnO-modified nanographene (MnO-NG) composite nanoparticles. The relevant precursor was supplied by Scharlau (Spain). The 99.9% pure nanographene (NG) used in the synthesis of composite nanoparticles was sourced from Nanokar Chemicals Industry and Trade Ltd. (Türkiye). According to the supplier specifications, the nanographene consists of few-layer graphene with micrometer-scale lateral dimensions, and these properties were verified in this study by the characteristic (002) X-ray diffraction (XRD) reflection, high BET surface area, and preserved aromatic carbon framework observed in the FTIR analysis.

### 2.2 Synthesis of MnO-Nanographene Composite Nanoparticles

The MnO-nanographene (MnO-NG) composite nanoparticles were prepared based on the conventional impregnation method. First, within the scope of this process, 0.88 g of Mn­(NO_3_)_2_·4H_2_O was completely dissolved in 100 mL of deionized water to achieve a MnO loading of 10% by weight. A 2.5 g amount of NG was added to the obtained clear solution, and the system was continuously stirred at room temperature for 24 h on a magnetic stirrer. After obtaining a homogeneous mixture, the mixture was heated to 100 °C to remove the solvent. The solvent was evaporated completely, after which the solid precursor material that remained was dried at 120 °C for 12 h. The solid that had been impregnated following the drying process was transferred to a tube furnace for heat treatment in conditions of inert atmosphere. The sample was heated to 550 °C under a nitrogen gas flow rate of 100 mL/min and kept at this temperature for 2 h.^18^ During this process, the Mn­(NO_3_)_2_·4H_2_O compound decomposed and reduced to MnO, resulting in the successful production of MnO-loaded NG (MnO-NG) composite nanoparticles. After the heat treatment was completed, the system was allowed to cool to room temperature. During the synthesis of MnO-NG, Mn^2+^ is adsorbed on the surface of NG to form hydroxyl manganese, which gradually decomposes to form MnO when heated at 550 °C.^19^

### 2.3 Green Synthesis of MnO-Nanographene/Green Composite Nanoparticles

In the green synthesis process of MnO-NG/green composite nanoparticles, hemp plant (*Cannabis sativa L*.) stalk parts were preferred as a bioextract source. The synthesis process includes sequential pretreatment steps and controlled reaction conditions. In the first stage, hemp stalks were dried at 80 °C for 24 h to remove moisture, then mechanically ground into fine particles, and subsequently sieved to a particle size of 300–500 μm. This obtained biomass was used for hydrothermal extraction. To achieve this, 25 g of sieved hemp stalks was placed in a 250 mL autoclave reactor containing 150 mL of deionized water. The reactor was then heated to 180 °C for 12 h. Once the reaction had finished, the system was cooled to room temperature, and the liquid phase was filtered through a 0.22 μm membrane filter to remove any solid residues. The obtained extract was stored in a refrigerator at 0–4 °C for use in further stages. Before producing the composite nanoparticles, 0.88 g of Mn­(NO_3_)_2_·4H_2_O was completely dissolved in 100 mL of the prepared hemp extract to achieve a 10% MnO content. Next, 2.5 g of NG was added to the mixture, which was then homogenized with a magnetic stirrer at room temperature for 24 h. The resulting suspension was concentrated by drying at 100 °C. The semisolid precursor material was then dried at 120 °C for 12 h to prepare it for the reaction. In the last stage, the dried impregnated material was placed in a tube-type oven and subjected to calcination treatment at 550 °C for 2 h under inert (N_2_ gas supplied with a flow rate of 100 mL/min) atmosphere conditions.^18^ The product was cooled to room temperature after being heated and was obtained as the final MnO-NG/green composite nanoparticles. All these synthesis steps are presented schematically in Figure 1.

**Figure 1:**
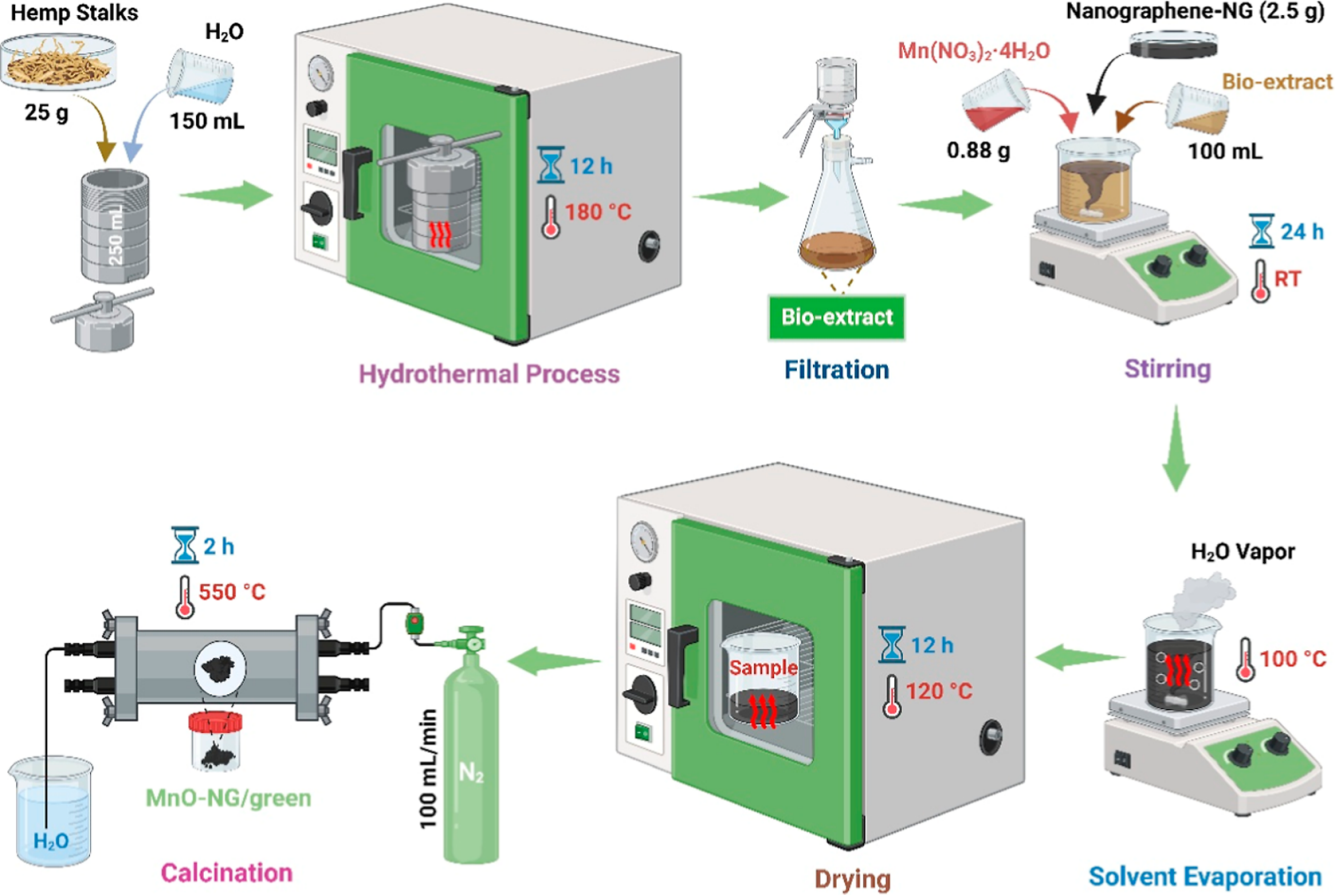
Schematic diagram showing the steps involved in preparing MnO-NG/green composite nanoparticles.

### 2.4 CO_2_ Uptake Process

To evaluate the CO_2_ capture performance of the developed MnO-NG and MnO-NG/green composite nanoparticles, experimental studies were conducted using the H-Sorb 2600 high-pressure gas adsorption analyzer, which has also been used in previous studies.^20,21^ Data collection and analysis were performed using the VxComm software interface, which operates in conjunction with the TDS-734 module.^21^ For each experiment, 0.25 g of composite nanoparticles was placed in the analysis tube. Before the experiment, the composite nanoparticles were predried under a vacuum at 100 °C for 24 h to remove moisture and volatile residues from the surface. Subsequently, the composite nanoparticles were held under a vacuum at 150 °C for 10 h to completely clean the surface and remove any residual gases. CO_2_ adsorption experiments were conducted at temperatures of 273 and 298 K and pressures ranging from 0 to 1 bar. This experimental procedure allows a detailed study of the CO_2_ uptake behavior of the prepared composite nanoparticles under different temperature and pressure conditions.

### 2.5 Composite Nanoparticle Characterization

Various characterization techniques were used to characterize NG, MnO-NG, and MnO-NG/green composite nanoparticles. The functional groups and chemical bonds of the composite nanoparticles were determined by Fourier transform infrared spectroscopy (FTIR, Bruker VERTEX 70v, USA). Crystal structure and phase analyses of the composite nanoparticles were carried out using an X-ray powder diffractometer (PANalytical Empyrean, Netherlands) with a Cu–Kα (λ = 1.5406 Å) beam in the range of 2θ = 10°–90°. A high-resolution scanning electron microscope (ZEISS Sigma 300, Germany) was used to examine the surface morphologies and microstructural features of the composite nanoparticles in detail. Additionally, both quantitative and qualitative elemental composition analysis of the composite nanoparticles was carried out using an energy dispersive X-ray spectroscopy (EDX) detector from Oxford Instruments integrated with the scanning electron microscopy (SEM) system. The analysis of the morphology, structure, and distribution of the composite nanoparticles was achieved by high-resolution images obtained by using a Hitachi High-Tech HT7700 transmission electron microscope (Japan). This analysis provided detailed information about the size, shape, and internal structure of the particles. The Brunauer–Emmett–Teller (BET) method using N_2_ adsorption isotherms performed at 77 K was used to evaluate the surface area and pore structure analyses of the composite nanoparticles. Quantachrome Quadrasorb SI (England) was used to perform these analyses. The determination of the pore size distribution was done by the Barrett–Joyner–Halenda (BJH) method, and the calculation of the total pore volume was performed according to nitrogen (N_2_) adsorption data at 0.99 RH. The t-plot method was used to determine the micropore volume.

## 3 Results and Discussion

### 3.1 Characterization of MnO-NG and MnO-NG/Green Composite Nanoparticles

The composite nanoparticles were structurally characterized using various analytical methods, including FTIR, XRD, SEM, transmission electron microscopy (TEM), EDX, and BET analyses.

#### 3.1.1 FTIR Analysis

The FTIR spectra of NG, MnO-NG, and MnO-NG/green composite nanoparticles are presented in Figure 2a. The characteristic peak observed at approximately 1545 cm^–1^ wavenumber in the NG nanoparticles corresponds to the stretching vibrations of aromatic C=C bonds,^22^ indicating that the carbon skeleton is preserved and the structure maintains its aromatic nature. However, the peak detected around 1136 cm^–1^ can be attributed to the stretching vibrations of C–O bonds.^23^ In addition, the peaks observed at 466 cm^–1^ and 520 cm^–1^ correspond to the vibration modes of Mn–O bonds.^24^

**Figure 2:**
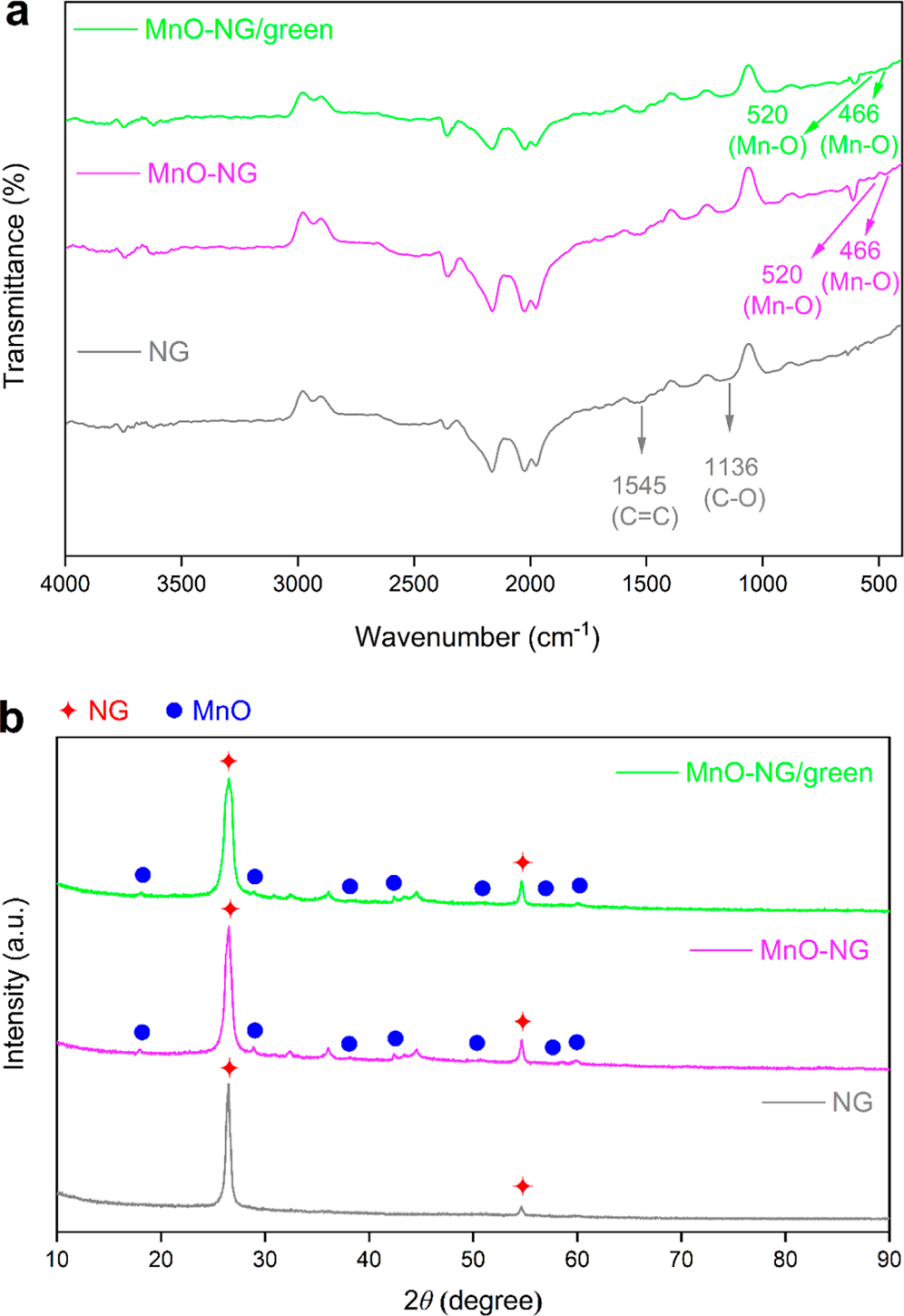
FTIR analysis results (a) and XRD diffraction patterns (b) of NG, MnO-NG, and MnO-NG/green composite nanoparticles.

#### 3.1.2 XRD Analysis

The XRD diffraction patterns of NG, MnO-NG, and MnO-NG/green composite nanoparticles are presented in Figure 2b. The NG nanoparticles exhibit a prominent (002) reflection at 2θ = 26.5°, indicating that they have higher crystallinity compared to the other samples.^25^ This (002) peak corresponds to the 2θ = 26.5° value, which is characteristic for graphite, and confirms the graphitic structure of NG.^26^ In addition, a prominent (002) peak at around 26.5° and a weak (004) peak around 54.6° are observed in the NG pattern, both reflections indicating a carbon-based structure.^25^ The XRD data of NG confirm that the crystalline structures specific to this material are consistent with previous literature studies.^25–27^ On the other hand, the sharp diffraction peaks determined at 2θ = 18.12°, 28.85°, 37.95°, 42.30°, 49.95°, 57.08°, and 60.13° angles in MnO-NG and MnO-NG/green composite nanoparticles, respectively, were matched with the (200), (310), (211), (301), (411), (600), and (521) planes, respectively, and it was determined that these patterns were compatible with JCPDS card no. 44-0141 for the MnO crystal structure.^28^ To further evaluate the structural characteristics of the MnO phase, the crystallite size of the MnO in the MnO-NG and MnO-NG/green composite nanoparticles was estimated using Scherrer’s equation (*K* = 0.9, Cu–Kα, λ = 1.5406 Å). The calculation was performed based on three intense MnO reflections at approximately 2θ = 28.85°, 37.95°, and 42.30° (JCPDS 44-0141). The average crystallite size was found to be 34.6 ± 4.4 nm for MnO-NG and 30.0 ± 10.1 nm for MnO-NG/green. The slightly smaller crystallite size observed for the green-synthesized sample is consistent with the TEM results, which revealed a more homogeneous dispersion and reduced agglomeration of MnO nanoparticles on the nanographene surface. The XRD patterns show that all of the characteristic diffraction peaks belonging to the MnO-NG and MnO-NG/green composites are consistent with the JCPDS 44-0141 card, clearly confirming the cubic MnO phase. The absence of additional peaks in the diffractograms corresponding to MnO_2_, Mn_2_O_3_, or unreacted precursor compounds indicates the phase purity of the synthesized structure. Furthermore, the calculation of the MnO crystallite size using the Scherrer equation, consistent with TEM results, supports more controlled nucleation and particle distribution, particularly in the green synthesis sample. These findings reveal that the biobased reduction medium directs the formation of MnO and limits the formation of impurities.

#### 3.1.3 SEM and TEM Analyses

The SEM and TEM images illustrating the morphological structure of NG, MnO-NG, and MnO-NG/green composite nanoparticles are presented in Figure 3. SEM and TEM images of MnO-NG composite nanoparticles synthesized by using the conventional method showed that the distribution of MnO nanoparticles on the NG surface was irregular and heterogeneous, and these particles showed local aggregation and clustering in some areas. Furthermore, it was determined that the nanoparticles were clearly agglomerated and formed irregular groups within the structure. This suggests that the conventional method provides limited control over the surface modification. In contrast, MnO-NG/green composite nanoparticles prepared using green synthesis were found to contain MnO nanoparticles with a more homogeneous, orderly, and dense distribution on the NG surface, as revealed by SEM and TEM analyses. This morphology indicates improved structural uniformity with significantly reduced agglomeration and more controlled binding of MnO to the NG surface. The green synthesis method appears to be superior to the conventional synthesis method in terms of morphological control and surface distribution. This is evidenced by the observed nanostructure of the MnO-NG/green composite nanoparticles obtained by green synthesis; this structure is more advantageous in terms of effective surface interactions.

**Figure 3:**
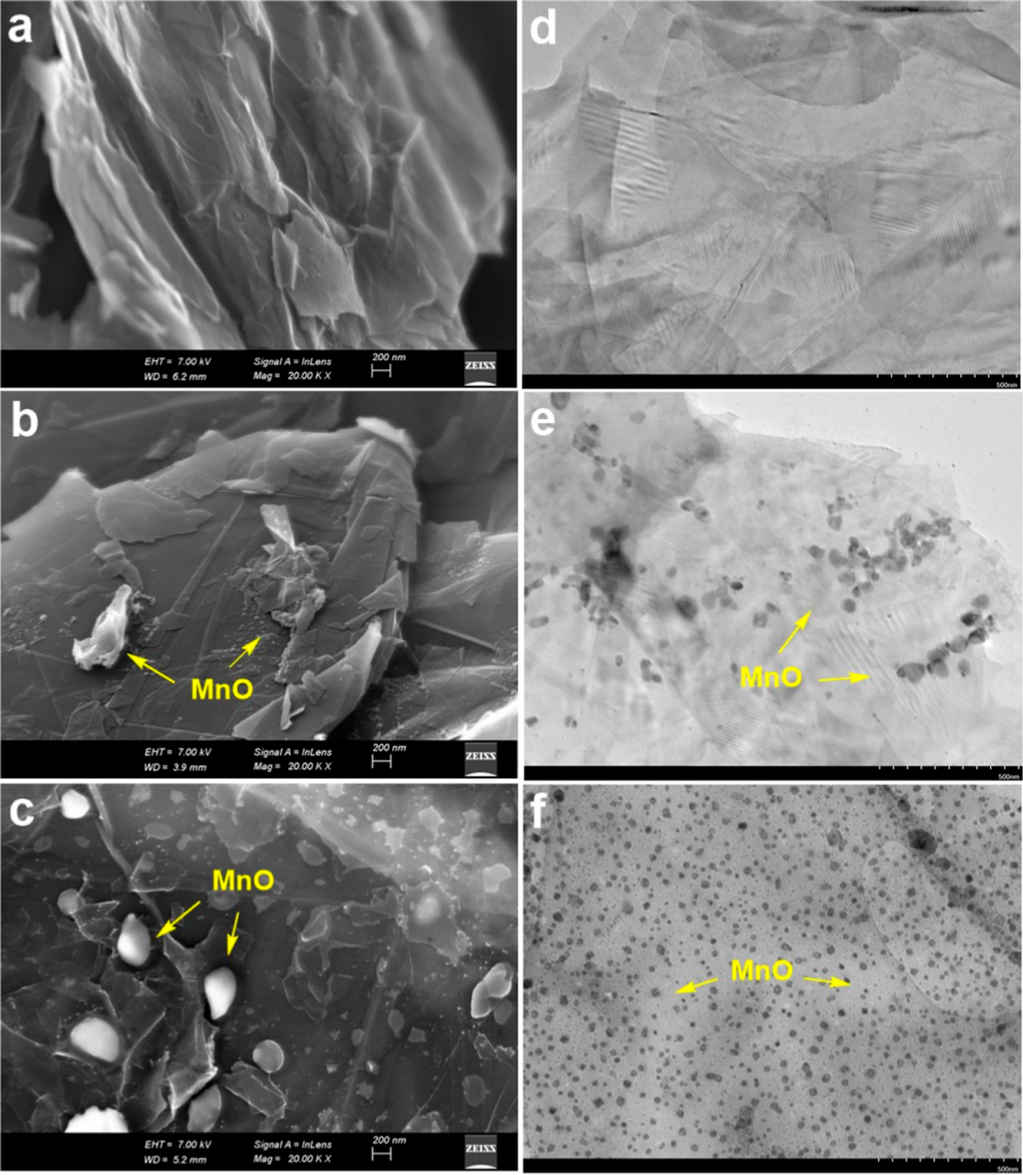
SEM and TEM images illustrating the morphological structure of NG, MnO-NG, and MnO-NG/green composite nanoparticles are presented in panels (a–d), (b–e), and (c–f), respectively.

#### 3.1.4 EDX Analysis

The EDX analysis results for the NG (a), MnO-NG (b), and MnO-NG/green (c) composite nanoparticles are presented in Figure 4. The distribution and percentage ratios of the elements on the composite surfaces are revealed by this analysis. The obtained data clearly show that the synthesized composites contain C, O, and Mn elements, as reported in previous studies.^29^ Semiquantitative analysis of EDX spectra confirmed the presence of C, O, and Mn elements in the MnO-NG and MnO-NG/green composites and determined that the Mn/C/O atomic ratios were consistent with the synthesized composite structure. In particular, it was observed that the Mn element exhibited a more balanced distribution on the surface of the MnO-NG/green sample. This finding supports the more homogeneous MnO distribution.

**Figure 4:**
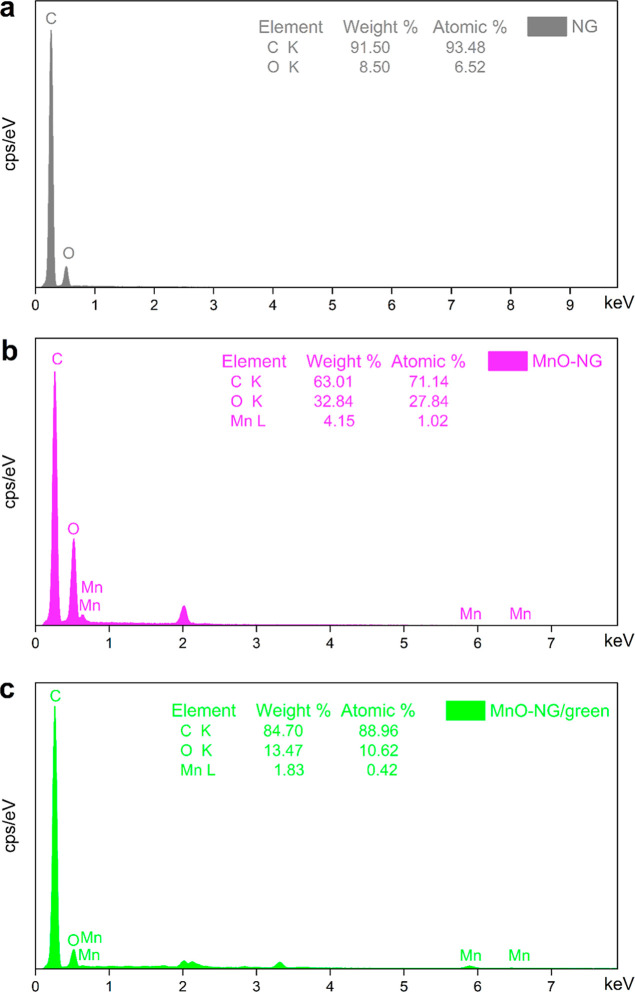
EDX analysis results for NG (a), MnO-NG (b), and MnO-NG/green (c) composite nanoparticles.

#### 3.1.5 BET Analysis

The N_2_ adsorption–desorption isotherms (a) and the BJH pore size distributions (b) of NG, MnO-NG, and MnO-NG/green composite nanoparticles are presented in Figure 5. The pore structure of the synthesized composites is directly evidenced by the N_2_ adsorption–desorption isotherms and the BJH pore size distribution curves. All three samples display a distinctive type IV isotherm profile with an H4-type hysteresis loop according to the International Union of Pure and Applied Chemistry (IUPAC) classification, confirming the predominance of mesoporous characteristics.^30,31^ The relatively narrow pore size distribution and the presence of slit-shaped pores are consistent with the layered structure of nanographene and the interparticle voids formed after MnO incorporation. The surface area and pore structure parameters obtained from N_2_ adsorption–desorption measurements are summarized in Table 1. The specific surface area (*S*_BET_) of the MnO-NG composite nanoparticle was measured at 702 m^2^/g, the total pore volume (*V*_Total_) was determined to be 0.45 cm^3^/g, the micropore volume (*V*_micro_) was found to be 0.19 cm^3^/g, the mesopore volume (*V*_meso_) was measured at 0.26 cm^3^/g, and the average pore diameter was calculated as 4.41 nm. By contrast, the respective values for the MnO-NG/green composite nanoparticles were found to be a *S*_BET_ of 629 m^2^/g, a *V*_Total_ of 0.40 cm^3^/g, a *V*_micro_ of 0.17 cm^3^/g, a *V*_meso_ of 0.23 cm^3^/g, and a pore diameter of 4.65 nm. These results further support the mesoporous framework and accessible pore network of the green-synthesized structure.

**Figure 5:**
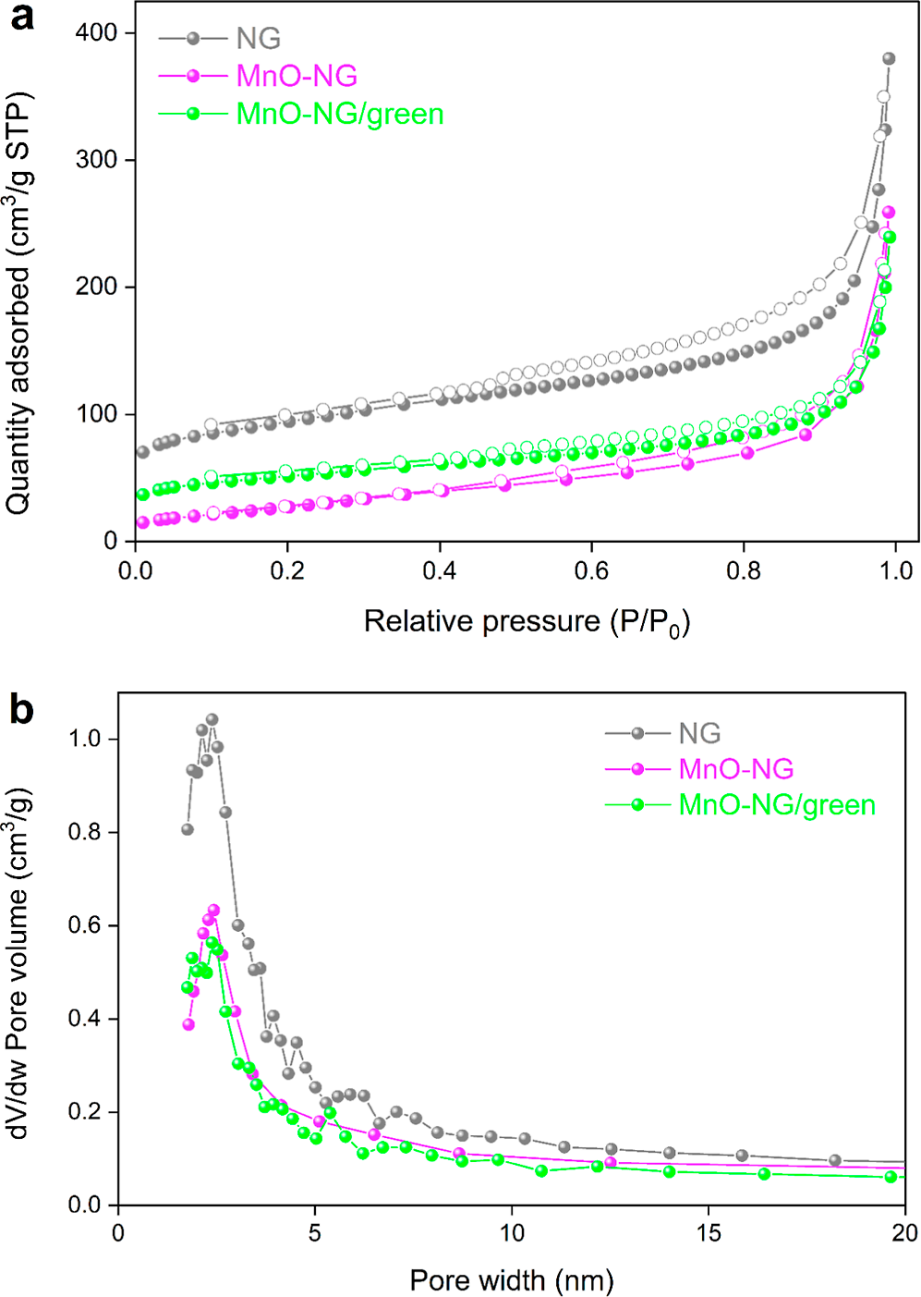
N_2_ adsorption–desorption isotherms (a) and BJH pore size distributions (b) of the NG, MnO-NG, and MnO-NG/green composite nanoparticles.

**Table 1:** Textural Properties of NG, MnO-NG, and MnO-NG/Green Composite Nanoparticles

textural properties\composites	NG	MnO-NG	MnO-NG/green
specific surface area (*S*BET, m2/g)	800	702	629
total pore volume (*V*_t_otal, cm3/g)	0.73	0.45	0.40
micropore volume (*V*micro, cm3/g)	0.26	0.19	0.17
mesopore volume (*V*meso, cm3/g)	0.47	0.26	0.23
pore diameter (nm)	3.00	4.41	4.65

### 3.2 CO_2_ Uptake Capacity of NG, MnO-NG, and MnO-NG/Green Composite Nanoparticles

The CO_2_ adsorption behaviors of NG, MnO-NG, and MnO-NG/green composite nanoparticles were systematically examined at temperatures of 298 and 273 K and in the pressure range of 0–1 bar. The CO_2_ adsorption isotherms of these materials at 298 and 273 K are presented in Figures 6a and 6b, respectively. The obtained isotherms show that the CO_2_ adsorption capacity increased significantly with an increasing pressure in all samples. In particular, the MnO-NG and MnO-NG/green composite nanoparticles exhibited an almost linear increase profile. The maximum CO_2_ uptake values obtained under 1 bar of pressure are quantitatively revealed in the comparative graph in Figure 6c. Within this framework, NG nanoparticles exhibit a CO_2_ adsorption potential of 2.07 mmol/g at 298 K and 2.59 mmol/g at 273 K. By contrast, the MnO-NG composite nanoparticle sample shows a significant increase to 3.74 mmol/g at 298 K and 4.43 mmol/g at 273 K under equivalent conditions. Exceptional performance was demonstrated by the MnO-NG/green composite nanoparticles with CO_2_ adsorption capacities of 4.94 mmol/g at 298 K and 5.81 mmol/g at 273 K. A clear trend was observed: an increase in the CO_2_ adsorption capacity as the temperature decreased for all composite nanoparticles. This confirms the role of physical adsorption at low temperatures on the binding of CO_2_ molecules to the surface and that these binding mechanisms become more effective at low temperatures.^32,33^

**Figure 6:**
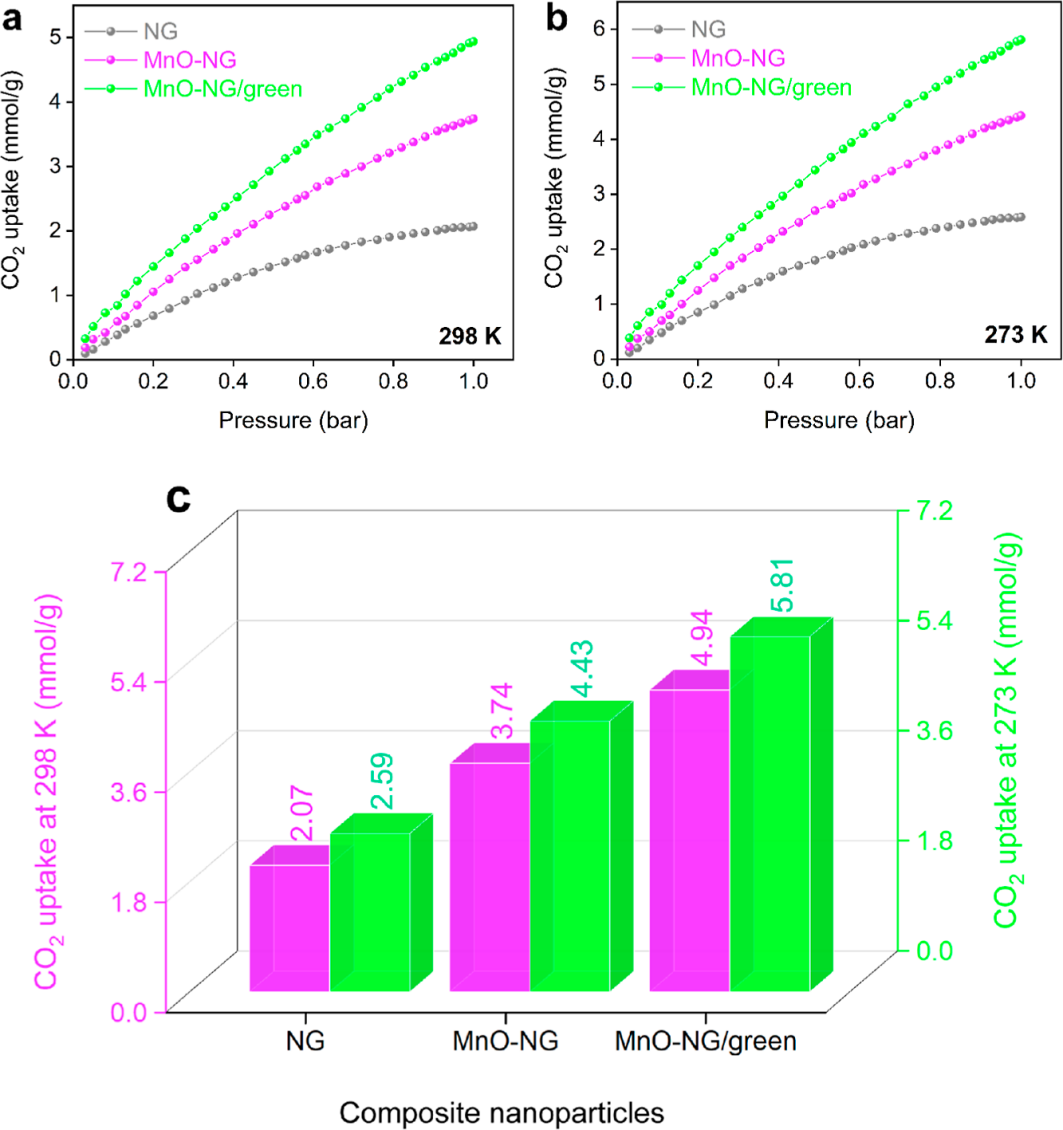
CO_2_ adsorption isotherms at 298 K (a) and 273 K (b) within the 0–1 bar pressure range and the CO_2_ uptake capacities at 298 and 273 K under 1 bar pressure (c) for NG, MnO-NG, and MnO-NG/green composite nanoparticles.

Although the MnO-NG/green composite has a lower specific surface area (629 m^2^/g) compared to the MnO-NG sample, it exhibits a higher CO_2_ adsorption capacity, indicating that adsorption performance cannot be explained solely by surface area.^30^ This is related to the distribution homogeneity of MnO nanoparticles and the accessibility of the active sites that they form on the surface. The hemp stalk extract used in the green synthesis process contributes to the more controlled formation of MnO nanoparticles by providing a reducing and stabilizing environment thanks to the phenolic compounds and oxygen-containing functional groups it contains.^17^ This controlled formation mechanism ensures that the nanoparticles are distributed more regularly and less agglomerated on the nanographene surface, thus allowing the Mn/O surface regions to be more open to interaction with CO_2_. Mn–O pairs on metal oxide surfaces can interact with the quadrupole moment of the CO_2_ molecule to form Lewis acid–base character binding sites.^15,16^ These interactions become more effective, particularly when homogeneous distribution is achieved, and increase the adsorption capacity. Furthermore, the contact regions formed at the graphene–MnO interface facilitate CO_2_ capture by supporting surface polarization and physical adsorption contributions.^32,33^ Therefore, the observed performance increase can be explained by the active site density, distribution regularity, and surface chemistry optimization, rather than the total surface area.

In this study, adsorption experiments were conducted using pure CO_2_ as a single-component gas, and this approach allows for a fundamental evaluation of the adsorbent’s surface interaction mechanism and active sites. In practical applications, however, CO_2_ is typically present alongside other components such as N_2_, CH_4_, and water vapor. The isosteric adsorption heat determined in the MnO-NG/green composite, ranging from 19.4 to 24.5 kJ/mol, indicates that adsorption is predominantly physical in nature and may therefore offer advantages in terms of regeneration in competitive gas environments and in the presence of moisture. Furthermore, the ability of Mn–O surface regions to interact with the quadrupole moment of the CO_2_ molecule indicates a preferential adsorption potential for CO_2_ compared to gases with lower polarity, such as N_2_ and CH_4_. In this context, the pure gas results serve as a fundamental indicator of the material’s behavior in realistic multicomponent gas environments.

### 3.3 Isotherm Modeling and Mechanism for CO_2_ Uptake by NG, MnO-NG, and MnO-NG/Green Composite Nanoparticles

The CO_2_ uptake mechanism was elucidated by fitting the widely used Langmuir and Freundlich isotherm models to the adsorption data obtained from experiments. The isotherms provide valuable information about the types of interactions occurring during the adsorption process, the structure of the adsorbent surface, and the properties of its active sites.^34^ The Langmuir model presented in eq 1 is based on the assumption of a homogeneous surface, where the energy of the active sites on the adsorbent surface is uniform and where adsorption occurs in a single layer. In contrast, the Freundlich model presented in eq 2 describes the multilayer adsorption behavior that occurs on heterogeneous surfaces, where the energy distribution is not uniform.^18^

1 $$q_{e}=\frac{{q_{m}K}_{L}P_{e}}{{1+K}_{L}P_{e}}$$

2 $$q_{e}=K_{F}{P_{e}}^{1/n_{F}}$$

where *q*_e_ (mmol/g) = adsorption capacity at equilibrium, *P*_e_ (bar) = partial pressure at equilibrium, *q*_m_ (mmol/g) = maximum adsorption capacity, *K*_L_ (1/bar) = Langmuir constant, *K*_F_ (mmol/g.bar ^1/*n*F^) = Freundlich constant, and the value of 1/*n* is an empirical constant that indicates surface heterogeneity. Adsorption is considered favorable when 0 < 1/*n* < 1.

The results obtained from the adsorption isotherm models (Figure 7 and Table 2) indicate that the experimental data for all samples are better represented by the Langmuir model. The model’s goodness-of-fit is confirmed not only by the high determination coefficient (*R* ^2^ > 0.99)^35^ but also by lower chi-square (χ^2^)^36^ and root-mean-square error (RMSE)^37^ values, as well as more negative Akaike Information Criterion (AIC)^38^ results. Furthermore, residual distribution plots showing a random distribution around zero indicate that the model is statistically reliable and does not contain a systematic bias. These findings suggest that adsorption occurs in a single layer on energetically similar active sites, indicating that the surface is largely homogeneous.

**Figure 7:**
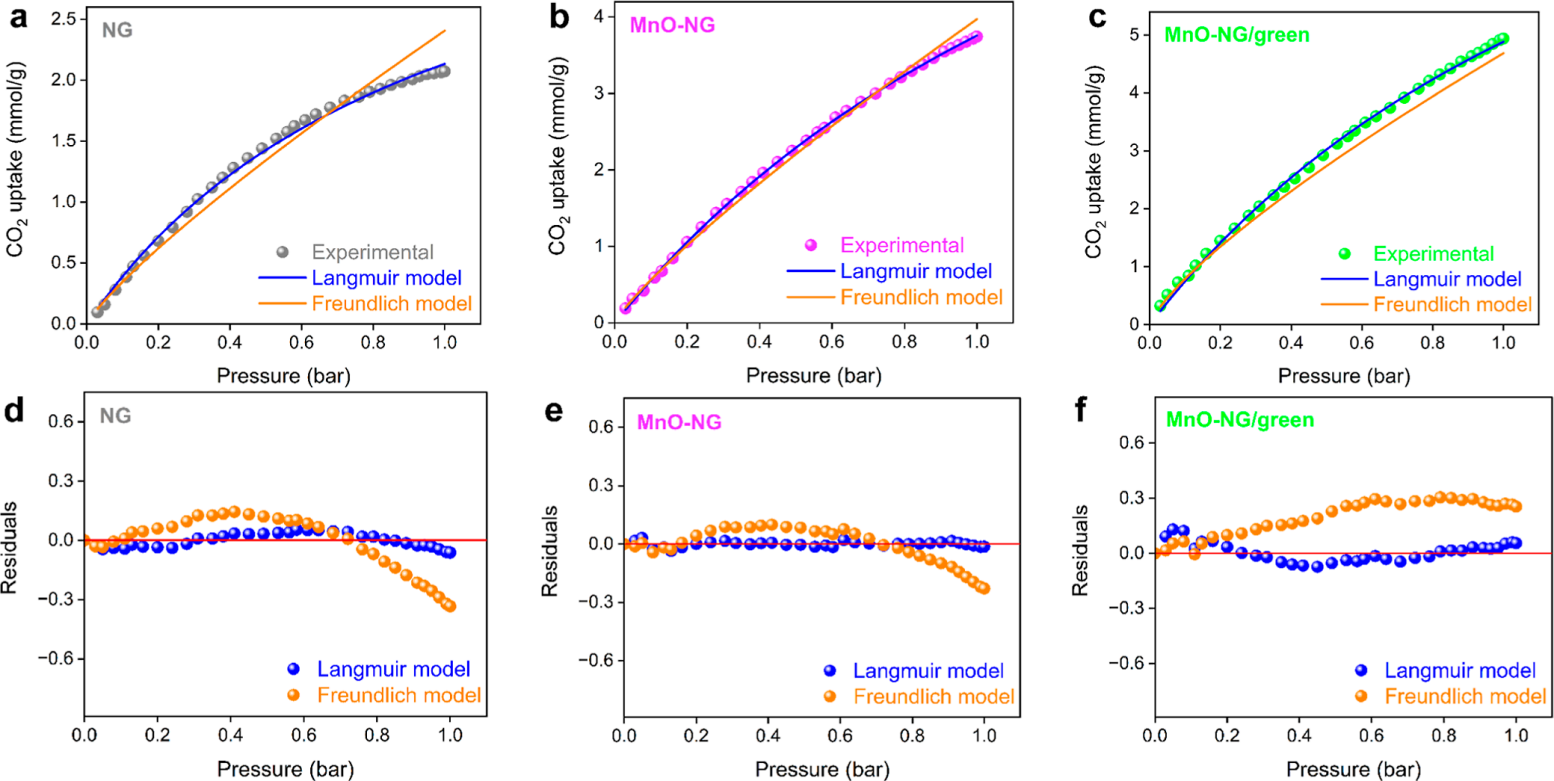
Plots of the fitting and residuals of the Langmuir and Freundlich isotherm models for CO_2_ uptake by NG (a–d), MnO-NG (b–e), and MnO-NG/green (c–f) composite nanoparticles at 298 K.

**Table 2:** Langmuir and Freundlich Isotherm Parameters and Statistical Error Analysis for CO_2_ Uptake by NG, MnO-NG, and MnO-NG/Green Composite Nanoparticles at 298 K

isotherm model	parameter	NG	MnO-NG	MnO-NG/green
Langmuir	*q*_m_ (mmol/g)	4.23	10.42	12.67
	*K*_L_ 1/(bar)	1.02	0.56	0.63
	*R* ^2^	0.997	0.999	0.998
	χ^2^	0.05	0.01	0.13
	RMSE	0.04	0.01	0.05
	AIC	–215.61	–278.91	–190.20
Freundlich	*K*_F_ (mmol/g.bar1/*n*F)	2.41	3.97	4.68
	*n*_F_	1.19	1.18	1.29
	*R* ^2^	0.954	0.993	0.978
	χ^2^	0.4	0.11	0.50
	RMSE	0.15	0.10	0.22
	AIC	–122.91	–150.03	–95.03

The combined evaluation of BET and isotherm analyses reveals that MnO nanoparticles are more homogeneously distributed in MnO-NG/green composites, thereby increasing the number of accessible active sites. The interaction of Mn–O surface species with the quadrupole moment of the CO_2_ molecule contributes to the formation of Lewis acid–base character binding sites, which increases the adsorption capacity. The calculated *Q*_st_ values (19.4–24.5 kJ/mol) are characteristic of physisorption, confirming that the adsorption process is primarily governed by reversible surface interactions. While the large π-electron surface of nanographene supports CO_2_ binding via dispersion forces, low diffusion barriers allow for the rapid transport of gas molecules. Consequently, it can be concluded that the MnO-modified surface forms regular active sites and that the graphene carrier structure facilitates gas diffusion.

Figure 8 schematically presents the proposed mechanism for the adsorption of CO_2_ on MnO-NG/green composite nanoparticles. The π-electron-rich basal plane of the graphene layer enables physical adsorption through dispersion forces and quadrupole–π interactions with CO_2_ molecules. In addition, Mn–O active pairs present in MnO nanoparticles, homogeneously distributed on the surface, contribute to the formation of Lewis acid–base interactions by facilitating the polarization of CO_2_ molecules. The more uniform MnO distribution obtained through the green synthesis approach increases the accessible active center density, supporting the formation of multiple adsorption sites and thus enhancing the CO_2_ holding capacity of the composite material. This mechanism is consistent with experimental isotherm results and structural characterization findings, revealing the decisive role of the synergistic interaction between MnO and nanographene in the adsorption performance.

**Figure 8:**
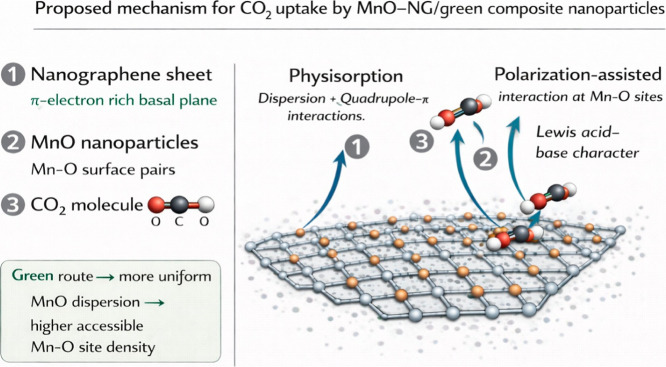
Schematic illustration of the proposed CO_2_ adsorption mechanism on MnO-NG/green composite nanoparticles.

### 3.4 Isosteric Heat of Adsorption for CO_2_ Uptake by NG, MnO-NG, and MnO-NG/Green Composite Nanoparticles

Isosteric heat of adsorption (*Q*_st_) is the measure of the heat energy given off by gas molecules that have been absorbed on the surface of the adsorbent, and this value provides valuable insights into the intensity and character of the interactions between the adsorbent and the adsorbate. Moreover, it is considered an important indicator of the reusability and thermodynamic stability of adsorbent materials.^32^ In the evaluation of CO_2_ adsorption in particular, isothermal adsorption heat (*Q*_st_) is a crucial parameter because it reveals the interaction between the gas and the material, helping to determine whether the adsorption is physical or chemical in natüre.^33^ In this study, the *Q*_st_ values for CO_2_ were calculated using the Clausius–Clapeyron equation based on isotherm data obtained at 273 and 298 K. This approach enabled a comparative evaluation of the interactions between CO_2_ molecules and NG, MnO-NG, and MnO-NG/green composite nanoparticles. The results are presented in Figure 9a. Experimental data indicate that MnO-NG/green composite nanoparticles possess a CO_2_ adsorption capacity ranging from 0.4 to 5.7 mmol/g, while the calculated *Q*_st_ values for this material vary between 19.4 and 24.5 kJ/mol. In MnO-NG composite nanoparticles, the adsorption capacity was determined to be in the range of 0.2–4.4 mmol/g, while the corresponding *Q*_st_ values were found to be between 18.8 and 23.9 kJ/mol. NG nanoparticles, on the other hand, exhibited the lowest CO_2_ capture performance, with an adsorption capacity ranging from 0.1 to 2.6 mmol/g and *Q*_st_ values ranging from 13.6 to 19.5 kJ/mol. MnO-NG/green composite nanoparticles differ from the other two nanomaterial types due to their higher CO_2_ adsorption capacity and isosteric enthalpy values. Despite the small decrease in the surface area of NG due to MnO modification, this indicates the formation of active sites on the surface that can interact more intensely with CO_2_. The *Q*_st_ range (19.4–24.5 kJ/mol) confirms that CO_2_ adsorption is dominated by physisorption, with moderate interaction strength that supports reversible adsorption and efficient regeneration. Conversely, the lower *Q*_st_ values of NG indicate a weaker interaction and lower binding energy with CO_2_ molecules. Consequently, the *Q*_st_ range determined for composite nanoparticles was found to be between 10 and 25 kJ/mol. CO_2_ adsorption is a predominantly physical process, involving interactions such as van der Waals forces.^32^ These findings clearly demonstrate the importance of surface chemistry and functional-group-enriched solid adsorbents as strategies to improve the CO_2_ capture performance.

**Figure 9:**
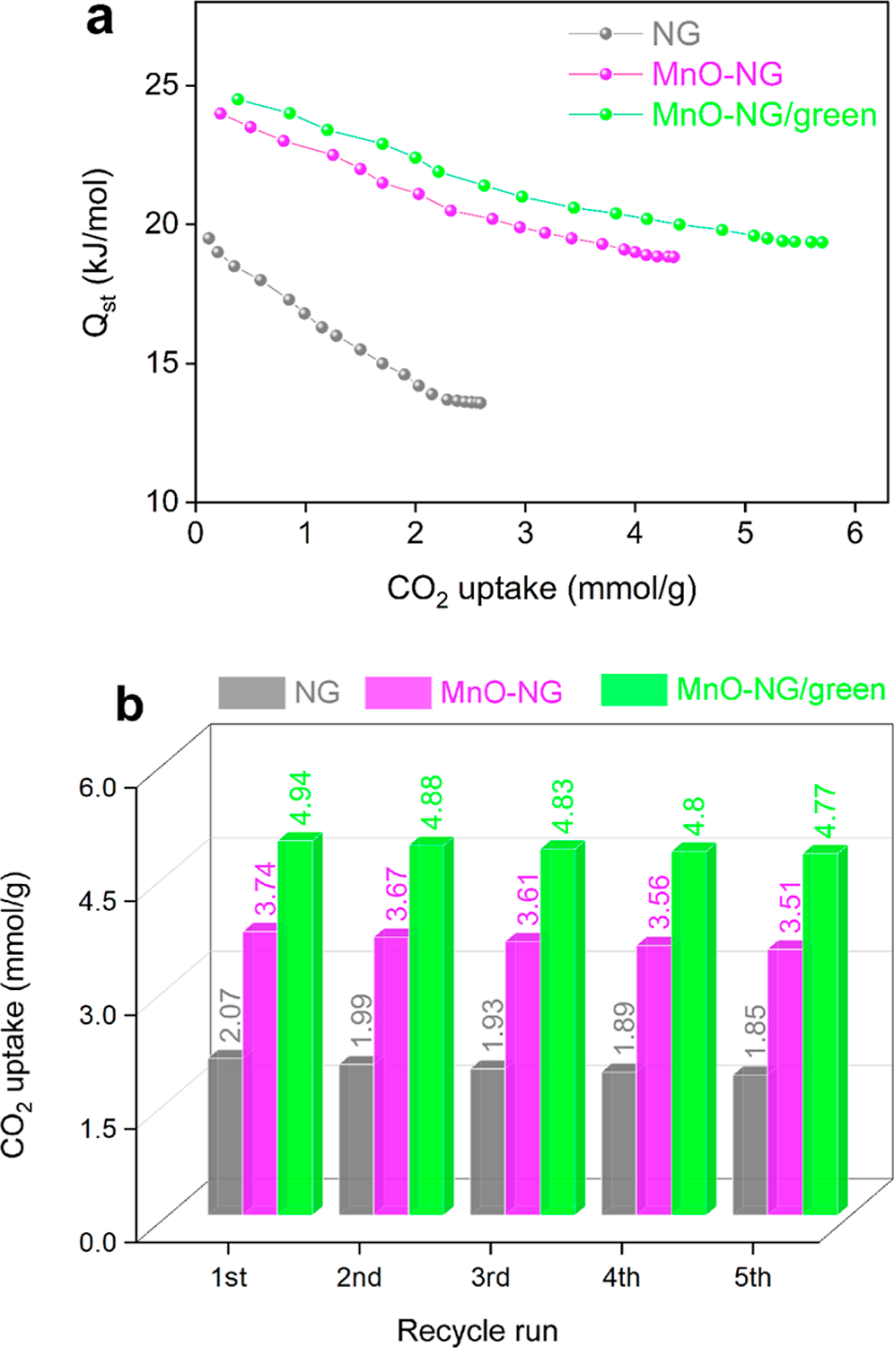
Isothermic heat of CO_2_ adsorption (a) and reusability performance during successive CO_2_ uptake cycles at 298 K and 1 bar (b) for NG, MnO-NG, and MnO-NG/green.

### 3.5 Reusability of NG, MnO-NG, and MnO-NG/Green Composite Nanoparticles for CO_2_ Uptake

The effectiveness of the adsorption process is largely dependent on the structural stability and reusability of the developed adsorbent across successive cycles. Therefore, the reusability performance of the synthesized NG, MnO-NG, and MnO-NG/green composite nanoparticles was thoroughly investigated through five consecutive adsorption–desorption cycles conducted at 298 K and 1 bar. Regeneration tests were performed following the adsorption process. In this stage, the composite nanoparticles were first held under a vacuum at 150 °C for 10 h to completely clean the surface and remove residual gas. Subsequently, the sample underwent a regeneration process under a constant flow of pure N_2_ at 110 °C for 2 h.^39^ The results are presented in Figure 9b. The experimental findings indicate that the reuse efficiency of the NG nanoparticles was approximately 89% after five cycles. Under the same conditions, the reuse efficiency was found to be 94% for MnO-NG and 97% for MnO-NG/green composite nanoparticles. These results demonstrate that the MnO-NG/green composite nanoparticles possess a very high regeneration capacity and largely maintain their structural integrity. The minimal decrease in the CO_2_ adsorption capacity observed across cycles indicates that this composite maintains its physical and chemical properties and is resistant to structural deformation. In this context, MnO-NG/green composite nanoparticles stand out as reusable adsorbents for CO_2_ capture applications thanks to their high durability, longevity, and reliability. While there is a slight decrease in capacity as the cycle progresses, the material’s high overall adsorption performance demonstrates its potential as a stable and effective CO_2_ capture agent, even under variable environmental conditions.

### 3.6 Comparison of CO_2_ Uptake Capacities of NG, MnO-NG, and MnO-NG/Green Composite Nanoparticles with Literature

Table 3 presents a comparative evaluation of the CO_2_ adsorption capacities of the composite nanoparticles synthesized in this study with various adsorbent materials previously reported in the literature. To make this comparison more comprehensive, Table 3 includes *Q*_st_ and reusability (cycle stability) data, where available, reported in the literature. This approach allows not only the CO_2_ uptake capacity but also the adsorption interaction strength and regeneration behavior to be evaluated together within the same framework. CO_2_ capture data obtained from experiments conducted at 298 and 273 K under a constant pressure of 1 bar were analyzed alongside literature findings under similar operating conditions. Such comparative analysis enables a more comprehensive interpretation of both the experimental parameters applied in the present study and the data available in the literature. As a result of the comparisons, it was observed that the synthesized composite structures exhibited performances compatible with or, in some cases, superior to similar systems reported in the literature in terms of the CO_2_ capture capacity. The CO_2_ adsorption capacities of NG, MnO-NG, and MnO-NG/green composite nanoparticles were determined to be 2.07, 3.74, and 4.94 mmol/g at 298 K, respectively, and 2.59, 4.43, and 5.81 mmol/g at 273 K, clearly demonstrating the effect of temperature on adsorption behavior. The MnO-NG/green composite nanoparticles obtained via green synthesis exhibited the highest adsorption performance among the evaluated structures. This suggests that the incorporation of the MnO component and the environmentally friendly synthesis route create accessible active surface sites that enable stronger and more specific interactions with the CO_2_ molecules. Furthermore, the experimental findings support the notion that MnO modification enhances the interaction potential with CO_2_ by improving the adsorbent surface properties.

**Table 3:** Comparison of CO_2_ Uptake Capacity, Isosteric Heat (*Q*_st_), and Reusability of Graphene-Based and Graphene–Metal Oxide Hybrid Adsorbents Reported in the Literature and in This Study

materials	temperature (K)	pressure (bar)	CO_2_ uptake (mmol/g)	*Q*_st_ (kJ/mol)	reusability	reference
graphene nanosheets	273	1	2.89	42.1	NR	^40^
GO-TiO2	298	1	1.88	19.6	NR	^41^
HKUST-1@10UV-GO	298	1	5.14	17.5	NR	^7^
KOH@GO-Fe_3_O_4_	298	9	6.81	NR	98% (5 cycles)	^42^
PEI-GO@ZIF-8	273	1	4.19	NR	NR	^43^
Pg@GO	300	7.88	4.80	NR	90% (8 cycles)	^44^
CTS/GO/ZnO composite	298	1	3.21	NR	95% (10 cycles)	^45^
rGO/NPC-600-2-1	298	1	5.77	NR	100% (10 cycles)	^46^
rGO-MnO_2_	273	1	6.03	43.6	100% (10 cycles); 90% (25 cycles)	^15^
MnO-NG/green	298	1	4.94	24.5	97% (5 cycles)	in this study
MnO-NG/green	273	1	5.81			

The “green synthesis” approach used in this study does not involve the complete elimination of the calcination step, which is performed at high temperatures. Thermal treatment at 550 °C is a necessary step in both conventional and green synthesis routes to obtain the crystalline MnO phase from the Mn­(NO_3_)_2_·4H_2_O precursor. However, this method is environmentally friendly due to the absence of toxic reducing agents and the use of an aqueous medium and a plant-derived extract that acts as a reducing and stabilizing agent. This reduces process safety and chemical hazard potential by eliminating the need for additional harmful chemicals. Although thermal processing requires energy input, the more homogeneous morphology and higher adsorption performance of the resulting material increase efficiency and offer a more environmentally balanced production approach.

## 4 Conclusions

This study provides a strong demonstration of the successful production of nanographene-manganese oxide (NG-MnO) composite nanoparticles via an environmentally friendly green synthesis method and of the use of this structure as a high-performance adsorbent for CO_2_ capture applications. Superior properties were exhibited by MnO-NG/green composites produced using bioextracts from the hemp plant when compared to MnO-NG structures synthesized via conventional methods, particularly in terms of morphological homogeneity, surface functionality, and adsorption performance. The MnO nanoparticles were found to be more uniformly and densely distributed on the NG surface in the MnO-NG/green composites that were obtained via green synthesis, as revealed by structural characterization analyses. BET analysis revealed that the MnO-NG/green composites had a highly developed porous structure, with a surface area of 629 m^2^/g and an average pore diameter of 4.65 nm. Although the MnO-NG/green composite has a lower BET surface area compared to that of the MnO-NG sample, it exhibits a higher CO_2_ adsorption performance, demonstrating that surface area alone is not the sole determining factor. Thanks to the green synthesis approach, MnO nanoparticles are distributed more homogeneously and controllably on the graphene surface; this has increased the accessibility of Mn–O active sites. Lewis acid–base interactions formed on the metal oxide surface and contact regions at the graphene–MnO interface have enabled stronger physical interactions with CO_2_, thereby increasing the adsorption capacity. These results indicate that the distribution of active sites and surface chemistry may be more important than the total surface area. CO_2_ adsorption experiments revealed that these composites had CO_2_ uptake capacities of 5.81 and 4.94 mmol/g at 273 and 298 K, respectively. The strong relationship between the synthesis method and structural properties is clearly demonstrated by the fact that these values are significantly higher than those of both pure NG and conventional MnO-NG. It was indicated by the analysis of the adsorption isotherm models that the experimental data were better fitted by the Langmuir model. Assessment of the thermodynamic nature of adsorption was via isosteric heat of adsorption (*Q*_st_) values, with the calculation of these values using the Clausius–Clapeyron equation. The *Q*_st_ values for the MnO-NG/green composites were found to be in the range of 19.4–24.5 kJ/mol. This indicates that the adsorption mechanism is predominantly characterized by physical adsorption (physisorption). Moreover, these composites’ structural stability and thermal resistance are confirmed by their 97% reuse efficiency after five consecutive adsorption–desorption cycles. Comparative assessments with existing literature have demonstrated that MnO-NG/green composites provide a CO_2_ capture potential that is equal to or better than that of numerous traditional and state-of-the-art adsorbents. It was also revealed by the study that surface interactions with CO_2_ are strengthened, and active surface areas that increase adsorption capacity are created by the homogeneous distribution of MnO on the graphene surface. In conclusion, the achievement of sustainable material production through a green synthesis approach with low environmental impact is not the study’s only accomplishment. The development of a high-performance, reusable, and environmentally friendly adsorbent that can contribute to the reduction of CO_2_ emissions is another. The resulting MnO-NG/green nanocomposites could be used in many different ways, from in the lab to in industry, which makes them a good choice for next-generation adsorbents in carbon capture technologies.

## Data Availability

The data that support the current study are available from the corresponding author upon reasonable request.
